# Supplementary material for: Content-rich biological network constructed by mining PubMed abstracts
Source: BMC Bioinformatics. 2004 Oct 8;5:147. doi: 10.1186/1471-2105-5-147 (PMC528731; doi:10.1186/1471-2105-5-147)
Supplement: Additional File 2 — The original results of the above study (non-essential files are deleted to keep the file size under the limit set by BMC bioinformatics). [file 1471-2105-5-147-S2.bz2 › chilibotAdditionalFile2/dip30/263ID7973658E1060/html/IL2RG_JAK3.html]

 


 **IL2RG** and **JAK3** 
  
Found 74 abstracts in PubMed, retrieved 30.  
 

 What does Google say? 
 PDF only 
| .edu only 

---

**Interactive relationship** (e.g. stimulation, inhibition, etc)

**Neutral relationship**- As a result of this interaction, p12 I increases signal transducers and activators of transcription 5 STAT5 DNA binding and transcriptional activity and this effect depends on the presence of both IL 2R beta and gamma c  [ **IL2RG** ]  chains and  **Jak3** .  Ref: 11468184 Blood, 2001
- It is generally held that the IL 2R is activated by the trans activity of the protein tyrosine kinases PTKs Jak1 and  **Jak3**  associated with beta c and gamma c  [ **IL2RG** ]  respectively.  Ref: 10889465 Cell Signal, 2000
- Such signals are likely relayed inside the cell by the tyrosine kinase  **Jak3** , which associates with gamma c  [ **IL2RG** ] .  Ref: 9850851 Immunol Rev, 1998
- The  **JAK3**  tyrosine kinase is constitutively associated with the gamma c  [ **IL2RG** ]  and is necessary for signaling through the gamma c  [ **IL2RG** ]  containing receptors.  Ref: 9801259 Semin Hematol, 1998
- Here we show that in normal lung fibroblasts IL 4 and IL 13 induce the expression of the gamma c  [ **IL2RG** ]  chain and its association with Janus kinase 3  [ **JAK3** ] , while lung myofibroblasts constitutively express a gamma c  [ **IL2RG** ]  chain displaying a limited association with  **JAK3** .  Ref: 12207328 Eur J Immunol, 2002
- Taken together, these results definitively demonstrate that IL 21 mediated signaling requires the gamma c  [ **IL2RG** ]  chain, and indicate that  **JAK3**  is an essential transducer of gamma c  [ **IL2RG** ]  dependent survival and or mitogenic signals induced by this cytokine.  Ref: 12093291 Biochemistry, 2002
- The Janus family tyrosine kinase 3  **Jak3**  is known to be associated with gamma c  [ **IL2RG** ] , and the reported phenotypes of gamma c  [ **IL2RG** ]  deficient gamma c  [ **IL2RG** ]  and  **Jak3**  deficient  **Jak3**  mice are similar, indicating that  **Jak3**  is an essential transducer of gamma c  [ **IL2RG** ]  dependent signals.  Ref: 10653847 Int Immunol, 2000
- Nonreceptor tyrosine kinase  **Jak3** , which binds to intracellular domain of interleukin 2 receptor gamma  [ **IL2RG** ]  subunit IL 2Rgamma, plays an important role in IL 2 function.  Ref: 12136181 Sheng Wu Hua Xue Yu Sheng Wu Wu Li Xue Bao Shanghai < MedlineTA>Sheng Wu Hua Xue Yu Sheng Wu Wu Li Xue Bao Shanghai , 2000
- Janus kinase 3  [ **JAK3** ]   **Jak3**  is essential for common cytokine receptor gamma chain gamma c  [ **IL2RG** ]  dependent signaling comparative analysis of gamma c  [ **IL2RG** ] ,  **Jak3** , and gamma c  [ **IL2RG** ]  and  **Jak3**  double deficient mice.  Ref: 10653847 Int Immunol, 2000
- Retroviral mediated transduction of wild type gamma c  [ **IL2RG** ]  into XSCID  [ **IL2RG** ]  JT cells restored function to the IL 21R, as shown by IL 21 induced tyrosine phosphorylation of JAK1 and  **JAK3** , and downstream activation of STAT5, in JT gamma c  [ **IL2RG** ]  cells as well as BaF3 IL 21R alpha and primary splenic B cells.  Ref: 12093291 Biochemistry, 2002
- Janus kinase 3  [ **JAK3** ]   **JAK3**  is a cytoplasmic tyrosine kinase required for T cell development and activated by cytokines that utilize the interleukin 2 IL 2 receptor common gamma chain gamma c  [ **IL2RG** ] .  Ref: 10380915 J Leukoc Biol, 1999
- No significant differences between cell lines were observed in IL 2 receptor chain alpha, beta, or gamma c  [ **IL2RG** ]  expression, in IL 2 mediated proliferation, or in IL 2 induced phosphorylated forms of Stat3, Stat5, Jak1, or  **Jak3** .  Ref: 10516077 J Virol, 1999
- These results indicate that  **Jak3**  is absolutely essential for gamma c  [ **IL2RG** ]  dependent T cell and B cell development, and for gamma c  [ **IL2RG** ]  dependent prevention of thymocyte apoptosis.  Ref: 10653847 Int Immunol, 2000
- In XSCID  [ **IL2RG** ]  and SCID resulting from mutations in  **JAK3** , which encodes a Janus family tyrosine kinase that couples to gamma c  [ **IL2RG** ]  and is required for gamma c  [ **IL2RG** ]  dependent signalling, T and natural killer NK cells are decreased but B cell numbers are normal T B NK SCID.  Ref: 9843216 Nat Genet, 1998
- Evidence from peripheral T cells indicates that survival signals arising from the intermediate affinity IL 2R lacking CD25 do not require the activation of Janus kinase 3  [ **JAK3** ]   **Jak3**  but do require the presence of the membrane proximal region of the gamma C  [ **IL2RG** ]  chain.  Ref: 12121224 Immunol Cell Biol, 2002

**Non-interactive relationship** (e.g. studied together, co-existance, homology, etc.)

- A  **Jak3**  independent signalling pathway involving p56 lck, generating anti apoptotic signals, can be observed and requires the PROX domain of gamma c  [ **IL2RG** ] .  Ref: 10889465 Cell Signal, 2000
- Interestingly, although Bcl 2 induction was previously suggested to be  **Jak3**  independent, IL 7 cannot induce Bcl 2 expression in CD4 single positive SP thymocytes in either gamma c  [ **IL2RG** ]  or  **Jak3**  mice nor can IL 7 rescue CD4 SP thymocytes from dexamethasone induced cell death in gamma c  [ **IL2RG** ]  or  **Jak3**  mice.  Ref: 10653847 Int Immunol, 2000
- Three critical events have been identified in the generation of the IL 2R signal for cell cycle progression, including heterodimerization of the cytoplasmic domains of the IL 2R beta and gamma c  [ **IL2RG** ]  chains, activation of the tyrosine kinase  **Jak3** , and phosphorylation of tyrosine residues on the IL 2R beta chain.  Ref: 9755337 Adv Immunol, 1998
- Defects in the gamma c  [ **IL2RG** ]  or  **Jak3**  result in T B severe combined immunodeficiency SCID.  Ref: 10573019 Eur J Hum Genet< MedlineTA>Eur J Hum Genet, 1998
- However, in EDgamma 16 cells, a gamma c  [ **IL2RG** ]  transfected ED40515 cell line, IL 21 binds to the IL 21R and can activate Janus kinase JAK 1,  **JAK3** , STAT1, and STAT3.  Ref: 11418623 J Immunol, 2001
- These data underline the pivotal role of the gamma c  [ **IL2RG** ]  chain, CD40 CD40L,  **JAK3**  and IL 13 in the inflammatory like activation of lung myofibroblasts, the cell type restraint effects of IL 13 on these cells, and the potential usefulness of  **JAK3**  inhibitors in the treatment of asthma.  Ref: 12207328 Eur J Immunol, 2002
- Immunoprecipitation and blotting of the LUst cell line with specific secondary antibodies demonstrated that the 140 kd phosphoprotein was the interleukin 4 receptor, the 130 kd phosphoprotein was Janus kinase 1, the 116 kd phosphoprotein was Janus kinase 3  [ **JAK3** ] , and the 65 kd phosphoprotein was the interleukin 2 receptor gamma chain  [ **IL2RG** ] .  Ref: 10612755 J Thorac Cardiovasc Surg, 2000
- The regulatory roles of the common cytokine receptor gamma chain gamma c  [ **IL2RG** ]  and  **Jak3**  dependent signaling in the proliferation and survival of mast cells were determined using gamma c  [ **IL2RG** ]  deficient gamma c  [ **IL2RG** ]  and  **Jak3**  deficient  **Jak3**  mice.  Ref: 10979963 Blood, 2000
- IL 7 signal transduction, involving gamma c  [ **IL2RG** ]  and  **Jak3** , is required for successful recombination at the TCR gamma locus.  Ref: 11404375 J Leukoc Biol, 2001
- Nevertheless, certain differences have been suggested related to the range of actions of gamma c  [ **IL2RG** ]  and  **Jak3** .  Ref: 10653847 Int Immunol, 2000
- Defects of the common gamma chain gamma c  [ **IL2RG** ]  or of the  **JAK3**  protein in humans have been shown to result in a severe combined immune deficiency SCID, with a profound defect in T and natural killer NK cell development.  Ref: 11213805 Immunol Rev, 2000
- T cells with wild type gamma c  [ **IL2RG** ]  and  **JAK3**  proteins have a profound selective advantage over cells that contain mutant proteins.  Ref: 9801259 Semin Hematol, 1998
- In the disease, gamma c  [ **IL2RG** ]  is expressed, but its binding to  **Jak3**  is reduced.  Ref: 12126929 Mol Genet Metab, 2002
- IL 4 phosphorylates the 65 kd isoform of Stat6 in mast cells in a gamma c  [ **IL2RG** ]  and  **Jak3**  dependent manner.  Ref: 10979963 Blood, 2000
- These results indicate that gamma c  [ **IL2RG** ]  and  **Jak3**  dependent signaling is essential for IL 4 and IL 9 induced proliferation and survival of murine mast cells, that the effects of IL 4 are mediated by type I IL 4R.  Ref: 10979963 Blood, 2000
- The common gamma chain gamma c  [ **IL2RG** ]  is a required signaling component of the IL 21 receptor and supports IL 21 induced cell proliferation via  **JAK3** .  Ref: 12093291 Biochemistry, 2002
- Moreover, we demonstrate a functional requirement for both gamma c  [ **IL2RG** ]  and the gamma c  [ **IL2RG** ]  associated Janus family tyrosine kinase 3  **JAK3**  in IL 21 induced proliferation of pro B lymphoid cells engineered to express human IL 21R alpha BaF3 IL 21R alpha.  Ref: 12093291 Biochemistry, 2002
- Although the mast cells in gamma c  [ **IL2RG** ]  and  **Jak3**  mice were morphologically indistinguishable from those in wild type mice, the number of peritoneal mast cells was decreased in gamma c  [ **IL2RG** ]  and  **Jak3**  mice as compared with that in wild type mice.  Ref: 10979963 Blood, 2000
- **Jak3** , a member of the Janus tyrosine kinase family is an intracellular kinase functionally coupled to cytokine receptors that share a common gamma chain gamma c  [ **IL2RG** ] .  Ref: 10573019 Eur J Hum Genet< MedlineTA>Eur J Hum Genet, 2000
- Defects of the gamma c  [ **IL2RG** ]   **JAK3**  signaling pathway as a model.  Ref: 11213805 Immunol Rev, 2000
- With the exception that T and B cells in  **Jak3**  mice express high levels of gamma c  [ **IL2RG** ] , the defects in thymocyte and peripheral T cell and B cell development are indistinguishable among gamma c  [ **IL2RG** ] ,  **Jak3**  and gamma c  [ **IL2RG** ]   **Jak3**  mice.  Ref: 10653847 Int Immunol, 2000
- In the latter cells, without exogenous cytokines, gamma c  [ **IL2RG** ]   **JAK3**  controls, through autocrine loops, tyrosine kinase 2 phosphorylation and the balance between functional and decoy high affinity receptors.  Ref: 12207328 Eur J Immunol, 2002
- While extensive molecular and biochemical analysis of these patients has been instrumental in understanding better the biological properties of the gamma c  [ **IL2RG** ]  and  **JAK3**  protein, an unexpected phenotypic heterogeneity of gamma c  [ **IL2RG** ]  and  **JAK3**  deficiency has emerged, indicating the need for appropriate and extensive investigations even in patients with atypical presentations.  Ref: 11213805 Immunol Rev, 2000
- However, the effects of IL 4 and IL 9 were absent in BMMCs from gamma c  [ **IL2RG** ]  and  **Jak3**  mice.  Ref: 10979963 Blood, 2000
- To clarify whether gamma c  [ **IL2RG** ]  dependent cytokines can partially transduce their signals without  **Jak3** , we compared lymphocyte development in gamma c  [ **IL2RG** ] ,  **Jak3** , and gamma c  [ **IL2RG** ]  and  **Jak3**  double deficient gamma c  [ **IL2RG** ]   **Jak3**  mice in the same genetic background.  Ref: 10653847 Int Immunol, 2000
- In addition, IL 4Ralpha, gamma c  [ **IL2RG** ] , and  **Jak3** , but not IL 2Rbeta or IL 7Ralpha, were expressed in BMMCs.  Ref: 10979963 Blood, 2000
- Role of common cytokine receptor gamma chain gamma c  [ **IL2RG** ]  and  **Jak3**  dependent signaling in the proliferation and survival of murine mast cells.  Ref: 10979963 Blood, 2000
- Furthermore, IL 4 phosphorylated the 65 kd isoform of Stat6 in BMMCs from wild type mice but not from gamma c  [ **IL2RG** ]  and  **Jak3**  mice.  Ref: 10979963 Blood, 2000
